# Calcium channel α2δ1 subunit is a functional marker and therapeutic target for tumor-initiating cells in non-small cell lung cancer

**DOI:** 10.1038/s41419-021-03522-0

**Published:** 2021-03-11

**Authors:** Yuanyuan Ma, Xiaodan Yang, Wei Zhao, Yue Yang, Zhiqian Zhang

**Affiliations:** 1 Department of Thoracic Surgery Unit II, Laboratory of Carcinogenesis and Translational Research (Ministry of Education/Beijing), Peking University Cancer Hospital and Institute, 52 Fucheng Road, Beijing, 100142 China; 2 Department of Cell Biology, Key Laboratory of Carcinogenesis and Translational Research (Ministry of Education/Beijing), Peking University Cancer Hospital and Institute, 52 Fucheng Road, Beijing, 100142 China

**Keywords:** Cancer stem cells, Predictive markers

## Abstract

It is hypothesized that tumor-initiating cells (TICs) with stem cell-like properties constitute a sustaining force to drive tumor growth and renew fully established malignancy. However, the identification of such a population in non-small cell lung carcinoma (NSCLC) has been hindered by the lacking of reliable surface markers, and very few of the currently available surface markers are of functional significance. Here, we demonstrate that a subpopulation of TICs could be specifically defined by the voltage-gated calcium channel α2δ1 subunit from non-small cell lung carcinoma (NSCLC) cell lines and clinical specimens. The α2δ1^+^ NSCLC TICs are refractory to conventional chemotherapy, and own stem cell-like properties such as self-renewal, and the ability to generate heterogeneous tumors in NOD/SCID mice. Moreover, α2δ1^+^ NSCLC cells are more enriched for TICs than CD133^+^, or CD166^+^ cells. Interestingly, α2δ1 is functionally sufficient and indispensable to promote TIC properties by mediating Ca^2+^ influx into cells, which subsequently activate Calcineurin/NFATc2 signaling that directly activates the expression of *NOTCH3*, *ABCG2*. Importantly, a specific antibody against α2δ1 has remarkably therapeutic effects on NSCLC xenografts by eradicating TICs. Hence, targeting α2δ1 to prevent calcium influx provides a novel strategy for targeted therapy against TICs of NSCLC.

## Introduction

Non-small cell lung cancer (NSCLC) represents ~85% of lung cancer, which is the leading cause of cancer-related mortality worldwide^1^. Despite aggressive frontline treatments including the use of target-specific small-molecule inhibitors, the prognosis of NSCLC is poor for most patients mainly due to its heterogenic nature, highly invasive potential and therapeutic resistance^1,2^. Tumor-initiating cells (TICs) are a subpopulation within a tumor that own stem cell-like properties such as self-renewal, differentiation and strong tumorigenic capacity^3,4^. These cells have the ability to give rise to the heterogeneous lineages of cancer cells that comprise the tumor, and hence are proposed to constitute a sustaining force to drive and maintain fully malignancy^5,6^. Therapeutic approaches targeting TICs may therefore provide promising strategies to improve the outcome of cancer therapy, including that of NSCLC^7^. In NSCLC, TICs have been isolated with markers such as CD133^8^, CD44^9^, or CD166^10^. However, some other studies have yielded conflicting results^11,12^, and very few of these markers are functionally linked to TIC properties. More robust markers that could be used for reliable identification of NSCLC TICs and preferentially serve as therapeutic targets are urgently needed.

We have previously identified that the isoform 5 of the voltage-gated calcium channel α2δ1 subunit (encoded by the gene CACNA2D1), which is specifically recognized by the monoclonal antibody (mAb) 1B50-1, is a functional marker and therapeutic target for TICs of hepatocellular carcinoma and small cell lung cancer^13,14^. The expression of α2δ1 results in calcium influx into cells, which subsequently determines the self-renewal, tumorigenic, survival and chemoresistance capabilities of TICs^13–15^. α2δ1 was known as an auxiliary subunit of voltage-gated calcium channel to mediate calcium influx into cells by functioning in both targeting and/or stabilization of the channel in the membrane and in shaping the specific gating properties of different channel isoforms including the channel-forming subunit α1^16,17^, although the calcium-independent function of α2δ1 was also appreciated in literature^18^. However, the calcium signaling pathways mediated by α2δ1 in the determination of TIC properties remain elusive.

As a second messenger, elevated intracellular calcium ions (Ca^2+^) result in the activation of calcium signaling, which regulates many cellular activities including cell migration, secretion, metabolism, and survival^19,20^. Among the key molecules involved in calcium signaling are the nuclear factor of activated T cells family members NFATc1, NFATc2, NFATc3, and NFATc4, which directly link calcium signaling to gene expression. They are located in the cytoplasm in a hyperphosphorylated state, and will subsequently translocate into nucleus to bind to the DNA-responsive elements (5′-GGAAA-3′) in target gene promoters to regulate gene transcription when they are dephosphorylated by calcineurin^21,22^. Ca^2+^ can activate Notch signaling pathway, a hallmark of TICs of many cancer types including NSCLC, and targeted therapy against Notch has been proved to suppress the TIC properties^12,23–25^, but how Notch signaling is activated in TICs is poorly understood.

Here, we show that α2δ1^+^ cells of NSCLC own the stem cell-like properties, and represent a subset with the highest tumorigenic potential, compared with those defined by CD133, or CD166. Moreover, higher expression of α2δ1 in cancer tissues was predictive of poor prognosis for patients with NSCLC. Interestingly, the expression of α2δ1 is sufficient and necessary for the acquisition and subsequent maintenance of TIC properties via Ca^2+^-Calcineurin/NFATc2-NOTCH3/ABCG2 signaling pathway. Importantly, mAb1B50-1 against α2δ1 can selectively eradicate TICs, providing a novel approach of targeted therapy for NSCLC.

## Results

### Identification of α2δ1 as a candidate for NSCLC TIC surface marker

**Fig. 1:**
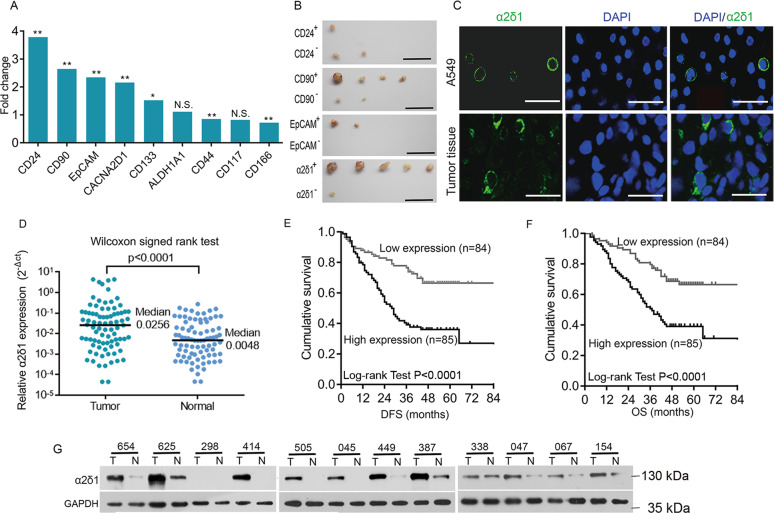
Identification of α2δ1 as a candidate marker for NSCLC TICs. **A** Transcriptome analysis shows the relative expression of the indicated molecules in NSCLC tissues including lung adenocarcinoma (*n* = 125) and squamous carcinoma (*n* = 224) compared with those in normal tissues (*n* = 54) from the TCGA database. *p ≤ 0.05, **p ≤ 0.001, n.s., no significance, *P* > 0.05, Mann–Whitney test. **B** Represented images showing the dissected tumors formed by 1000 cells per site of the indicated fractions purified by FACS from A549 cell line (n = 5). Scale bars, 2 cm. **C** Immunofluorescent staining for α2δ1 with mAb 1B50-1 in A549 cells and cryostat section of primary NSCLC tissue. Nuclei were stained with DAPI. Scale bar, 50 µm. **D** The relative expression levels of *α2δ1* to *GAPDH* mRNA in NSCLC tissues and paired normal tissues adjacent to tumors (*n* = 83) as measured by qRT-PCR. **E**, **F** Kaplan–Meier curves showing the disease-free survival (DFS) and overall survival (OS) of the patients with high and low levels of *α2δ1* mRNA in NSCLC tissues, which were divided according to the cutoff of 0.0155, the median value of α2δ1 relative to *GAPDH* mRNA. **G** Western blot results showing the expression of α2δ1 protein in 12 pairs of fresh NSCLC (T) and adjacent normal (N) tissues.

In view of the fact that many of the TIC markers were overexpressed in tumor tissues^10,26,27^, we performed data mining in the TCGA transcriptome database to identify candidates for surface markers of NSCLC TICs, which were overexpressed in the cancer tissues of NSCLC. Compared with that in the normal tissues, the expression of *CD24*, *CD90*, *EpCAM*, *CACNA2D1*, and *CD133* was found highly expressed in the cancer tissues (Fig. 1A). Flow cytometry results showed that the percentage of positive cells of the abovementioned molecules varied greatly across NSCLC cell lines including A549, H520, H292, H1299, PC9, H157, and GLC82, ranging from <1% to almost 100% (Supplementary Table 1). We further tested the tumorigenic potential of fluorescence-activated cell sorting (FACS)-purified positive and negative populations of the abovementioned molecules from the lung adenocarcinoma cell line A549 by subcutaneously (s.c.) transplanting 1000 and 100 cells of each subset in Nonobese diabetic/severe combined immunodeficient (NOD-SCID) mice. Although all the CD90^+^, EpCAM^+^, and α2δ1^+^ A549 subpopulation showed higher tumorigenic capabilities than their negative counterparts, the TIC frequency of α2δ1^+^ cells is the highest among these subpopulations tested (Fig. 1B, Supplementary Table 2). Hence, α2δ1 was selected for further characterization as a potential TIC surface marker for NSCLC.

### Clinical significance of α2δ1 expression in NSCLC patients

Consistently with its localization in cultured A549 cells, α2δ1 localized in the cell membrane of α2δ1^+^ cells, which were sparsely distributed in the cancer tissues as demonstrated by immunofluorescent staining (Fig. 1C). The expression of *α2δ*1 mRNA in cancer tissues was significantly higher than that of matched normal tissues as detected in 83 paired NSCLC and adjacent normal tissues by quantitative reverse transcription-polymerase chain reaction (qRT-PCR) (Fig. 1D). Further analysis of the expression *α2δ1* mRNA in 169 cases of NSCLC tissues has revealed that the expression of *α2δ1* mRNA correlated positively with metastasis and advanced TNM stages of these patients, although it was not found significantly correlated with age, smoking history, gender, or venous invasion (Supplementary Table 3). Kaplan–Meier curves revealed that higher *α2δ*1 expression in cancer tissues was significantly associated with shorter disease-free survival and overall survival of these NSCLC patients (Fig. 1E, F). Multivariate COX regression analysis also demonstrated that higher α2δ1 mRNA level was an independent risk factor of unfavorable prognosis for these patients (Supplementary Table 4). The overexpression of α2δ1 in NSCLC was also confirmed at the protein level by Western blot in 12 paired cancer and adjacent normal tissues (Fig. 1G).

### Confirmation of α2δ1 as a TIC marker for NSCLC

**Fig. 2:**
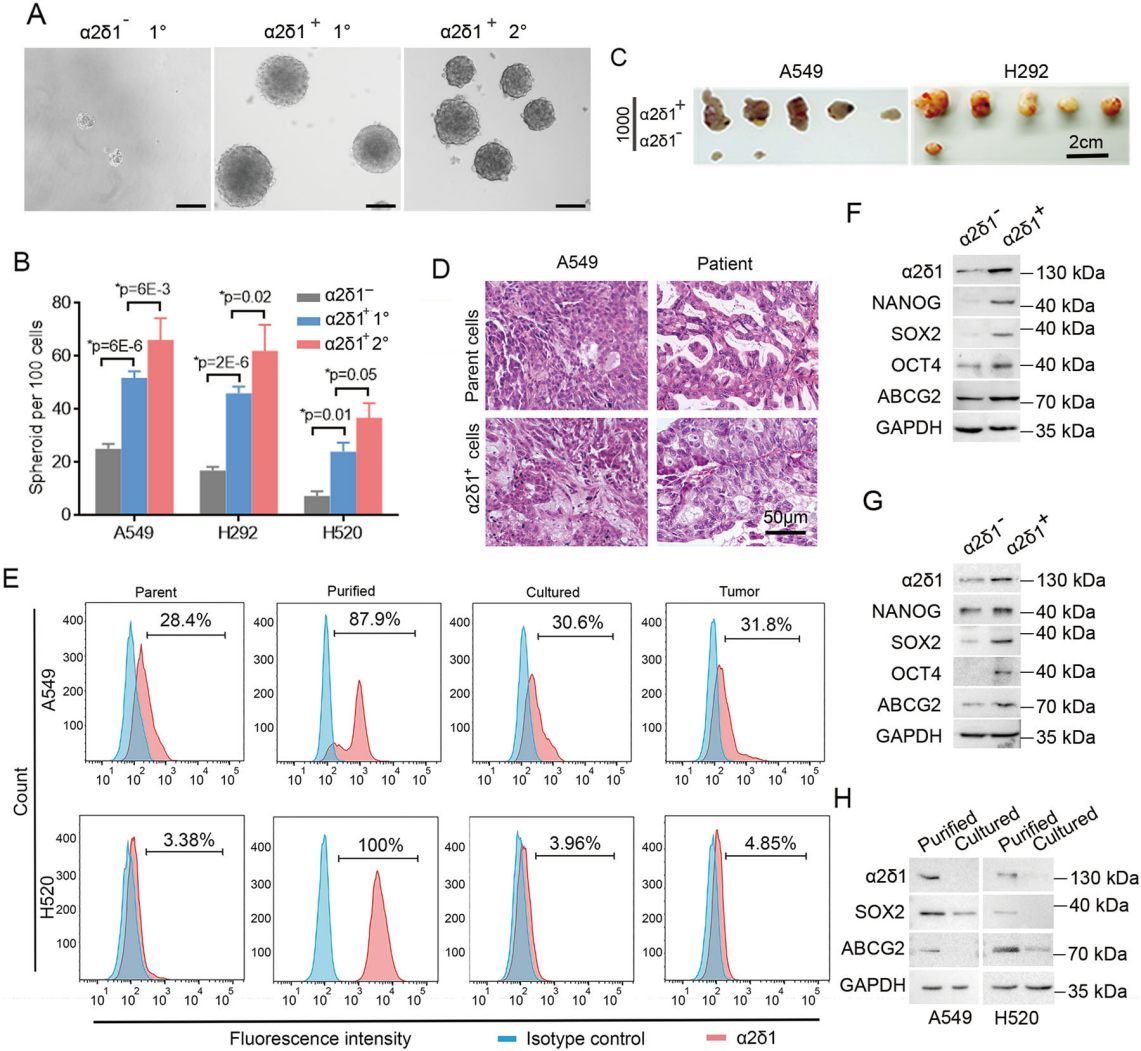
The stem cell-like properties of α2δ1^+^ NSCLC cells. **A** Representative phase-contrast photographs showing the primary (1°) and serially passaged (2°) spheroids formed by indicated subpopulations sorted from H520 cell line by FACS. Scale bars, 100 µm. **B** Histograms show the primary (1°) and serially passaged (2°) spheroid formation efficiencies of the α2δ1^+^ cells as compared with α2δ1^–^ cells sorted from indicated sources. Data are presented as the mean ± SD of three independent experiments (*n* = 3). *Student’s *t*-test. **C** Photographs of dissected tumors formed by sorted α2δ1^+^ and α2δ1^–^ cells from indicated sources (*n* = 5). **D** The histology of the tumors formed by α2δ1^+^ cells sorted from the A549 cell line and the primary tumor of one NSCLC patient was compared with that of the parent A549 tumor and the original patient tumor, respectively, by H.E. staining. **E** Flow cytometry analysis of the percentage of α2δ1^+^ fractions in parental cells (Parent), freshly FACS-purified α2δ1^+^ subpopulations (Purified) and purified α2δ1^+^ cells cultured in 10% FBS for 2 weeks (Cultured) or engrafted into NOD/SCID mice (Tumor). **F**–**H** The expression of the indicated molecules in purified α2δ1^+^ and α2δ1^–^ cells from A549 (**F**), H520 (**G**) cells lines, as well as in sorted α2δ1^+^ A549 and H520 fractions cultured in FBS-containing medium for two weeks (**H**) was analyzed by Western blotting. GAPDH serves as loading control.

To verify that α2δ1 indeed marks a population of TICs of NSCLC with stem cell-like properties, we sorted both the α2δ1^+^ and α2δ1^−^ subsets from the NSCLC cell lines A549, H520 and H292 using FACS to assay their spheroid-formation abilities in serum-free medium first. Purified α2δ1^+^ cells were able to form spheres at much higher rates than their negative counterparts (Fig. 2A, B). Moreover, single cells dissociated from these spheroids could be clonally expanded and subsequently passaged with increased sphere-forming efficiencies, indicating that the α2δ1^+^ cells in these NSCLC cell lines own the in vitro self-renewal capacity.

Next, we serially transplanted purified α2δ1^+^ and α2δ1^-^ cells from these cell lines into NOD/SCID mice s.c. with limiting dilution to test their tumor-initiation ability. Sorted α2δ1^+^ subsets from A549, H520 and H292 cell lines were sufficient to initiate tumor formation in almost all the transplanted mice, whereas no significant tumor formation was observed for most of the mice transplanted with α2δ1^−^ counterparts (Fig. 2C, Table 1). Further sorting and re-transplanting of the α2δ1^+^ subsets from the tumors formed by the α2δ1^+^ cells revealed that these cells were again able to generate tumors in the secondary recipient mice (Table 1). The tumorigenic capabilities of the α2δ1^+^ and α2δ1^−^ cells were further tested using purified fractions from fresh tumor tissues of NSCLC. As few as 100 α2δ1^+^ cells purified from tumor tissues of 4 cases out of 5 patients tested were enough to initiate tumor growth in most of the transplanted mice, whereas only tiny nodules were observed in 2 out of 34 mice transplanted with α2δ1^−^ cells (Table 1). Hematoxylin and eosin staining revealed that the histological features of tumors formed by α2δ1^+^ cells are morphologically heterogeneous, resembling those of the tumors from which they originated (Fig. 2D). These results indicate that α2δ1^+^ NSCLC cells are able to drive the formation of heterogeneity of tumors and own the in vivo self-renewal capability.

**Table 1:** The tumorigenicity of α2δ1^+^ and α2δ1^−^ cells of NSCLC in NOD/SCID mice.

Tumor cells	α2δ1	Tumor formation		Frequency of tumorigenic cells (95% CI)	*P* value
		1000	100		
A549	Negative	2/5	0/5	1/2213 (1/8821–1/555)	2.60E−05^c^
	Positive	5/5	4/5	1/62 (1/185–1/21)	
	ST	5/5	4/5	1/62 (1/185–1/21)	
H292	Negative	1/5	0/5	1/4983 (1/35191–1/706)	2.28E−06^c^
	Positive	5/5	4/5	1/62.1 (1/185–1/21)	
	ST	5/5	4/5	1/62.1 (1/185–1/21)	
H520	Negative	1/5	0/5	1/4983 (1/35191–1/706)	0.0383^c^
	Positive	3/5	2/5	1/ 711 (1/1928–1/263)	
	ST	5/5	3/5	1/109 (1/348–1/34)	
7494^a^	Negative	0/3	0/3	N.A.	
	Positive	3/3	3/3	N.A.	
9157^a^	Negative	0/3	0/3	N.A.	
	Positive	0/3	0/3	N.A.	
0296^a^	Negative	0/1	0/1	N.A.	
	Positive	1/1	0/1	N.A.	
7470^b^	Negative	1/5	0/5	1/4983 (1/35191–1/706)	0.09^c^
	Positive	3/5	1/5	1/921 (1/2617–1/324)	
9613^b^	Negative	1/5	0/5	1/4983 (1/35191–1/706)	0.038^c^
	Positive	3/5	2/5	1/711 (1/1928–1/263)	

*N.A*. not applied, The calculation of TIC frequencies is not applicable because there are too few animals transplanted, *ST* serial transplantation.

^a^Primary NSCLC tissue.

^b^NSCLC PDX tissues.

^c^Compared between the α2δ1^+^ and α2δ1^−^subpopulations.

We subsequently assessed the differentiation potential of the purified α2δ1^+^ cells both in vitro and in vivo. After sorted α2δ1^+^ cells from A549 and H520 cell lines were cultivated in a medium containing 10% FBS for two weeks, the percentages of α2δ1^+^ fractions reduced from more than 87% to those similar to the parent cell lines (Fig. 2E). Furthermore, the α2δ1^+^ percentage in the tumors formed by α2δ1^+^ subsets also decreased to 31.8% and 4.85% for A549 and H520, respectively (Fig. 2E). The data demonstrate that α2δ1^+^ NSCLC cells are able to differentiate into α2δ1^−^ cells.

Finally, the expression of stem cell-related factors such as NANOG, OCT4, SOX2 and ABCG2 was dramatically higher in the sorted α2δ1^+^ fractions of A549 and H520 cell lines than their negative counterparts. As additional evidence to support that α2δ1^+^ NSCLC cells have the capacity of differentiation, the expression of stem cell-related molecules including α2δ1, SOX2, and ABCG2 was also downregulated in purified α2δ1^+^ A549 and H520 fractions after cultivation in FBS-containing medium for 2 weeks (Fig. 2H).

Collectively, the above data demonstrate that α2δ1 defines a subset of NSCLC TICs with stem cell-like properties.

### The α2δ1^+^ NSCLC cells are resistant to conventional chemotherapy

**Fig. 3:**
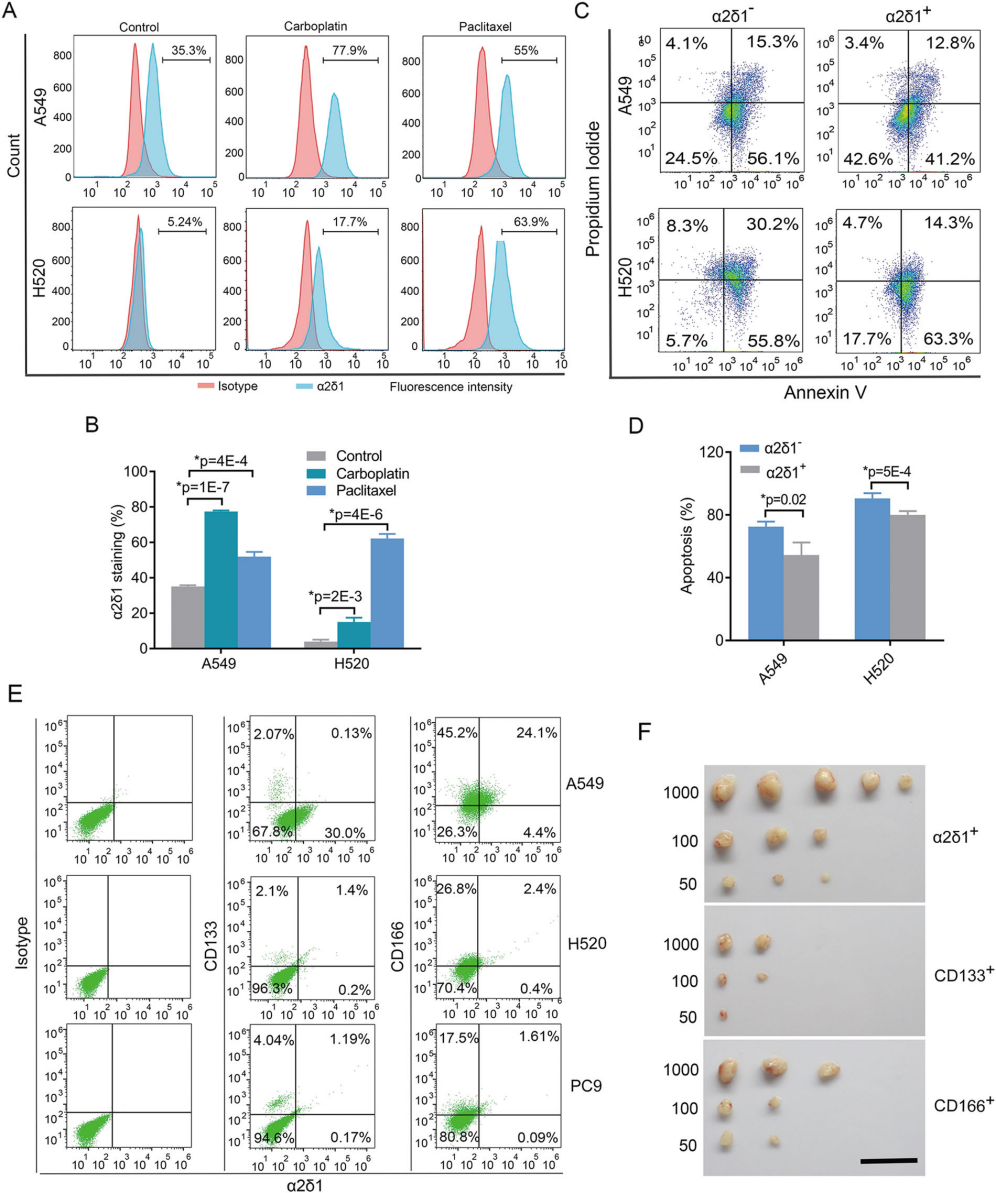
Characterization of α2δ1^+^ TICs of NSCLC. **A** Representative flow cytometry analysis of α2δ1^+^ fractions following carboplatin (10 µmol/L) and paclitaxel (1 nmol/L) treatment for 96 h. **B** Histograms showing the change of the percentage of α2δ1^+^ cells after carboplatin and paclitaxel treatment. **C** Representative flow cytometry analysis of the apoptosis of both the α2δ1^+^ and α2δ1^–^ cells from indicated sources after carboplatin treatment for 48 h at 10 µ mol/L as measured by flow cytometry. **D** Histograms showing the percentage of apoptotic cells of indicated subsets upon carboplatin treatment. **E** The relationship of the expression of α2δ1 with CD133 and CD166 as detected by flow cytometry in the indicated NSCLC cell lines. **F** Side-by-side comparison of the tumorigenic potential of α2δ1^+^ cells with that of CD133^+^ and CD166^+^ A549 cells by limited dilution transplantation into NOD/SCID mice (*n* = 5 for each group). Scale bar, 2 cm. The error bars in (**B**) and (**D**) represent SD of three independent experiments. *Student’s *t-test*.

To address whether α2δ1^+^ TICs of NSCLC are resistant to conventional chemotherapy, we first detected the ratio change of α2δ1^+^ TICs in the cell lines A549 and H520 after treatment with carboplatin and paclitaxel. The α2δ1^+^ TICs were significantly enriched after carboplatin and paclitaxel treatment in the two cell lines (Fig. 3A, B). Moreover, the apoptotic rates of α2δ1^+^ TICs were remarkably lower than their negative counterparts with the treatment of carboplatin (Fig. 3C, D). These data indicate that α2δ1^+^ TICs are indeed resistant to conventional chemotherapy drugs.

### TICs are more enriched in α2δ1^+^ cells than in CD166^+^, or CD133^+^ ones

We then sought to determine the correlation between α2δ1 and CD133, or CD166, the two widely used NSCLC TIC markers, by dual-color flow cytometry. Although the majority of α2δ1^+^ cells were positive for CD133 in the NSCLC cell lines H520 and PC9, very few α2δ1^+^ cells were positive for CD133 in the A549 cell line. Conversely, <40% of CD133^+^ cells were positive for α2δ1 in all the cell lines tested (Fig. 3E). The data indicate that there is no definite correlation existed between α2δ1 and CD133 in NSCLC cell lines. However, more than 84% of α2δ1^+^ cells were clearly CD166 positive but not vice versa in these cell lines (Fig. 3E), suggesting that α2δ1^+^ cells are a subset of CD166^+^ NSCLC cells.

To further address which marker is the best to identify NSCLC TICs, we performed a side-by-side comparison of the tumorigenic potential among α2δ1^+^, CD133^+^, and CD166^+^ A549 cells in NOD-SCID mice. In consistent with the results reported in literature^10^, we did find the tumorigenic potential of CD166^+^ cells was slightly stronger than that of CD133^+^ ones. However, the tumor-initiation ability of α2δ1^+^ cells is the highest among these population (Fig. 3F, Supplementary Table 5), indicating that α2δ1 is the most robust marker for enriching NSCLC TICs compared with CD133 and CD166.

### The function of α2δ1 in the acquisition and maintenance of TIC properties

**Fig. 4:**
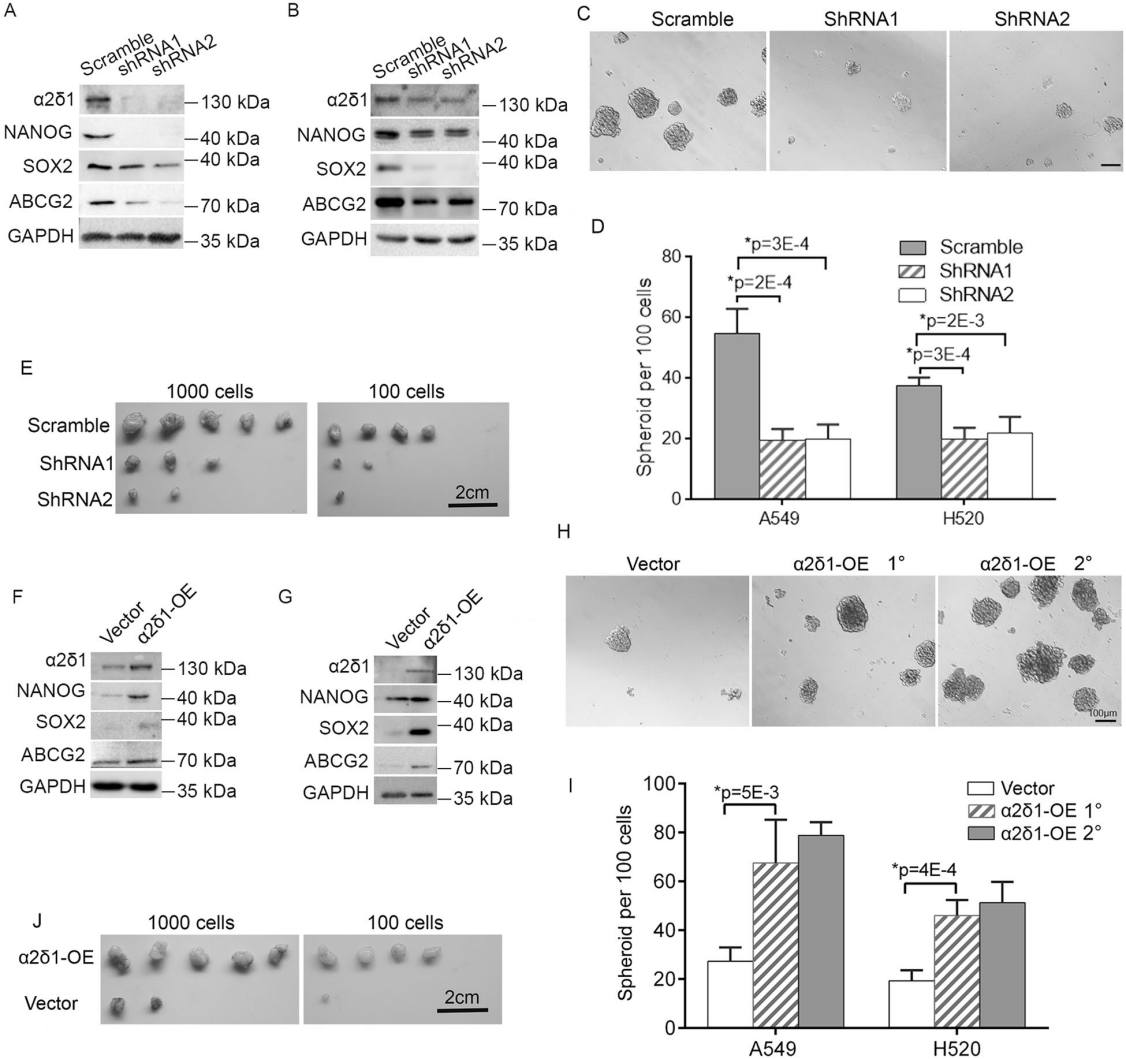
The roles of α2δ1 in the determination of NSCLC TIC’s properties. **A**, **B** Western blot results showing the effects of α2δ1 knockdown on the expression of indicated molecules in the sorted α2δ1^+^ subsets from the NSCLC cell lines A549 (**A**) and H520 (**B**). **C** Representative phase-contrast micrographs demonstrating the effect of α2δ1 knockdown on the spheroid formation of α2δ1^+^ A549 cells. Scale bar, 100 μm. **D** Histograms showing the spheroid forming efficiencies of the α2δ1^+^ subsets from indicated sources after α2δ1 knockdown with shRNAs (*n* = 4). **E** The effect of α2δ1 knockdown on the tumor-initiation ability of α2δ1^+^ A549 cells (*n* = 5). **F**, **G** Western blot analyses of the expression of indicated molecules in A549 (**F**) and H520 (**G**) cells overexpressing α2δ1. (**H**) Representative phase-contrast micrographs showing the spheroids formed by A549 cells overexpressing α2δ1. **I** Histograms showing the primary (1°) and subsequently passaged (2°) spheroid forming efficiencies of indicated cells overexpressing α2δ1 (*n* = 4). **J** Photograph of dissected tumors showing the tumor-initiating ability of α2δ1^−^ A549 cells overexpressing α2δ1 by limiting dilution transplantation in NOD-SCID mice (*n* = 5). The error bars in (**D**) and (**I**) represent SD of three independent experiments. *Student’s *t*-test. OE overexpression.

To test if α2δ1 play any roles in the acquisition and subsequent maintenance of the properties of NSCLC TICs, we performed both loss-of-function and gain-of-function studies in NSCLC cell lines. We first infected FACS-purified α2δ1^+^ subsets of A549 and H520 cell lines with lentiviruses harboring shRNAs against α2δ1 to address whether α2δ1 is required for the maintenance of TIC properties. Compared with the cells infected with lentivirus harboring scramble shRNA, α2δ1 knockdown resulted in downregulation of stem cell-related genes such as NANOG, SOX2, and ABCG2 (Fig. 4A, B), as well as remarkable inhibition of both the spheroid formation efficiencies and the tumorigenic potential of these α2δ1^+^ cells (Fig. 4C–E, Table 2).

**Table 2:** The tumorigenicity of indicated cells with α2δ1 knockdown or overexpression.

Group	Tumor fromation		Frequency of Tumorigenic cells (95% CI)	*P* value
	1000	100		
α2δ1^+^ A549 Scramble	5/5	4/5	1/62 (1/185–1/21)	
α2δ1 shRNA1	3/5	2/5	1/712 (1/1928–1/263)	0.002
α2δ1 shRNA2	2/5	1/5	1/1445 (1/4728–1/442)	9.63E–05
α2δ1^+^ H520 Scramble	5/5	2/5	1/184 (1/609–1/56)	
α2δ1 shRNA1	2/5	0/5	1/2212 (1/8821–1/555)	0.0031
α2δ1 shRNA2	3/5	0/5	1/1268 (1/3956–1/407)	0.0159
α2δ1^−^ A549 Vector	2/5	1/5	1/1445 (1/4728–1/442)	
α2δ1-OE	5/5	4/5	1/62 (1/185–1/21)	9.63E–05
α2δ1^−^ H520 Vector	2/5	0/5	1/2212 (1/8821–1/555)	
α2δ1-OE	4/5	2/5	1/452 (1/1205–1/170)	0.0428

*OE*, overexpression.

We next overexpressed α2δ1 in the cell lines A549 and H520 to test if α2δ1 is adequate to enable α2δ1^−^ cells to acquire stem cell-like phenotypes. Ectopic expression of α2δ1 in these α2δ1^−^ cells led to upregulation of a series of stem cell-related genes tested including NANOG, SOX2, and ABCG2 (Fig. 4, F, G), as well as significantly enhanced ability to initiate spheroid formation and to expand in subsequent serial propagation when grown in serum-free medium (Fig. 4, H, I), and increased tumorigenic capacity in NOD/SCID mice (Fig. 4J, Table 2).

These data demonstrate that α2δ1 is sufficient to reprogram α2δ1^−^ cells into TICs and is essential for the maintenance of the TIC properties of α2δ1^+^ NSCLC cells.

### The role of α2δ1 in driving TIC capacities is Notch-dependent

**Fig. 5:**
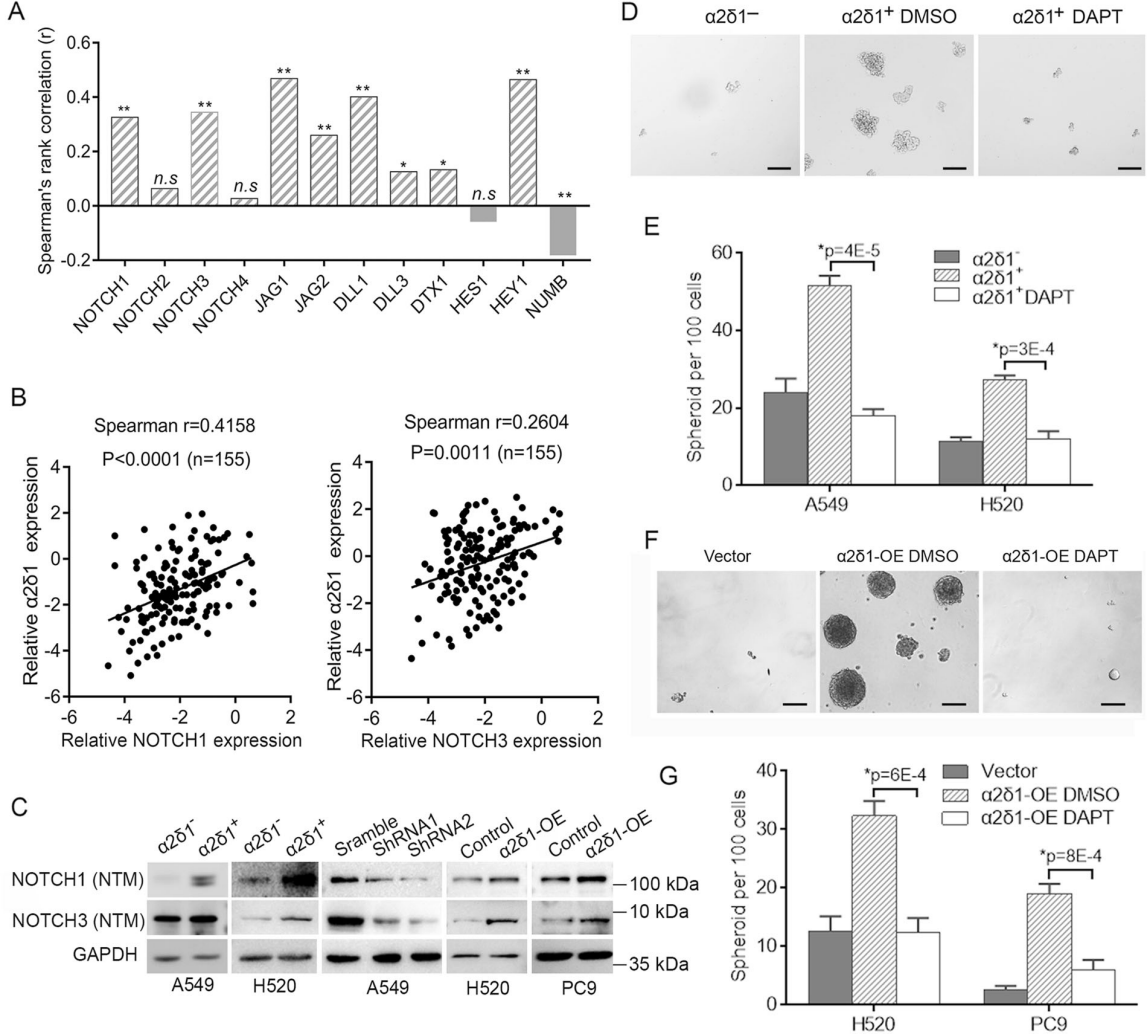
The roles of α2δ1 in driving TIC capacities is dependent on Notch signaling. **A** Histograms show the correlation of *α2δ1* mRNA with the genes related to Notch signaling in NSCLC tissues (*n* = 349) from TCGA database. *p ≤ 0.05, **p ≤ 0.001, n.s.>0.05. **B** The correlation between the expression of *α2δ1* and *NOTCH1*, and *NOTCH3* was detected by qRT-PCR in clinical NSCLC samples. **C** Western blot analysis of the expression of NOTCH1 and NOTCH3 in both the α2δ1^+^ and α2δ1^–^ subsets sorted from the indicated sources, purified α2δ1^+^ A549 cells with α2δ1 knockdown by shRNAs, and the indicated cells overexpressing α2δ1. **D** Phase-contrast photographs showing the spheroids formed by α2δ1^−^, or the α2δ1^+^ A549 cells treated with vehicle control DMSO or DAPT (10 µmol/L). Scale bars, 100 µm. **E** Histograms showing the spheroid formation efficiencies of the purified α2δ1^−^, or α2δ1^+^ cells from indicated sources treated with vehicle control DMSO or DAPT (10 µmol/L) (*n* = 3). **F** Representative phase-contrast micrographs demonstrating the spheroids formed by H520 cells overexpressing α2δ1 following DAPT treatment. Scale bars, 100 µm. **G** Histograms showing the spheroid forming efficiencies of the indicated cells overexpressing α2δ1 treated with vehicle control DMSO or DAPT (*n* = 3). The error bars in (**E**) and (**G**) represents SD of three independent experiments. *Student’s *t*-test.

To test if Notch signaling pathway associate with the α2δ1^+^ TICs of NSCLC, we first determined the correlation between the expression of α2δ1 and those genes involved in Notch signaling in NSCLC tissues from TCGA database. The expression of α2δ1 was found positively correlated with *NOTCH1* and *NOTCH3*, but not *NOTCH2* and *NOTCH4*, of the four Notch universal receptors, and their ligands *JAG1*, *JAG2*, *DLL1*, *DLL3* (Fig. 5A). The positive correlation between the expression of *α2δ1* and *NOTCH1*/*NOTCH3* was further validated in 155 cases of NSCLC specimens by qRT-PCR (Fig. 5B). We then checked the expression of NOTCH1 and NOTCH3 in the sorted α2δ1^+^ and α2δ1^−^ fractions from A549 and H520 cell lines. Compared with that in α2δ1^−^ subsets, the expression of NOTCH1 and NOTCH3 receptor was much higher in α2δ1^+^ populations purified from both cell lines as demonstrated by Western blotting (Fig. 5C). Forced expression of α2δ1 in H520 and PC9 cell lines resulted in elevated expression of NOTCH3, and minor increase of NOTCH1 at protein level. Conversely, knockdown of α2δ1 in purified α2δ1^+^ cells from A549 cell line led to downregulation of both NOTCH1 and NOTCH3 (Fig. 5C). These data indicate that α2δ1 can regulate the expression of NOTCH3 at least.

We subsequently treated sorted α2δ1^+^ TICs and the α2δ1-overexpressing cells with a γ-secretase inhibitor, DAPT, to address if NOTCH was functionally required for the in vitro self-renewal capacity of TICs mediated by α2δ1 using spheroid formation assay. Treatment of α2δ1^+^ TICs purified from A549 and H520 cell lines resulted in significant suppression of their spheroid formation ability (Fig. 5D, E). Furthermore, when the H520 and PC9 cells with forced expression of α2δ1 were treated with DAPT, the spheroid formation ability mediated by α2δ1 was dramatically attenuated (Fig. 5F, G).

Taken together, these data demonstrate that Notch signaling is essential for the role of α2δ1 in the acquisition and subsequent maintenance of the self-renewal property of NSCLC TICs.

### α2δ1 upregulates NOTCH3 through Ca^2+^-Calcineurin/NFATc2 signaling pathway

**Fig. 6:**
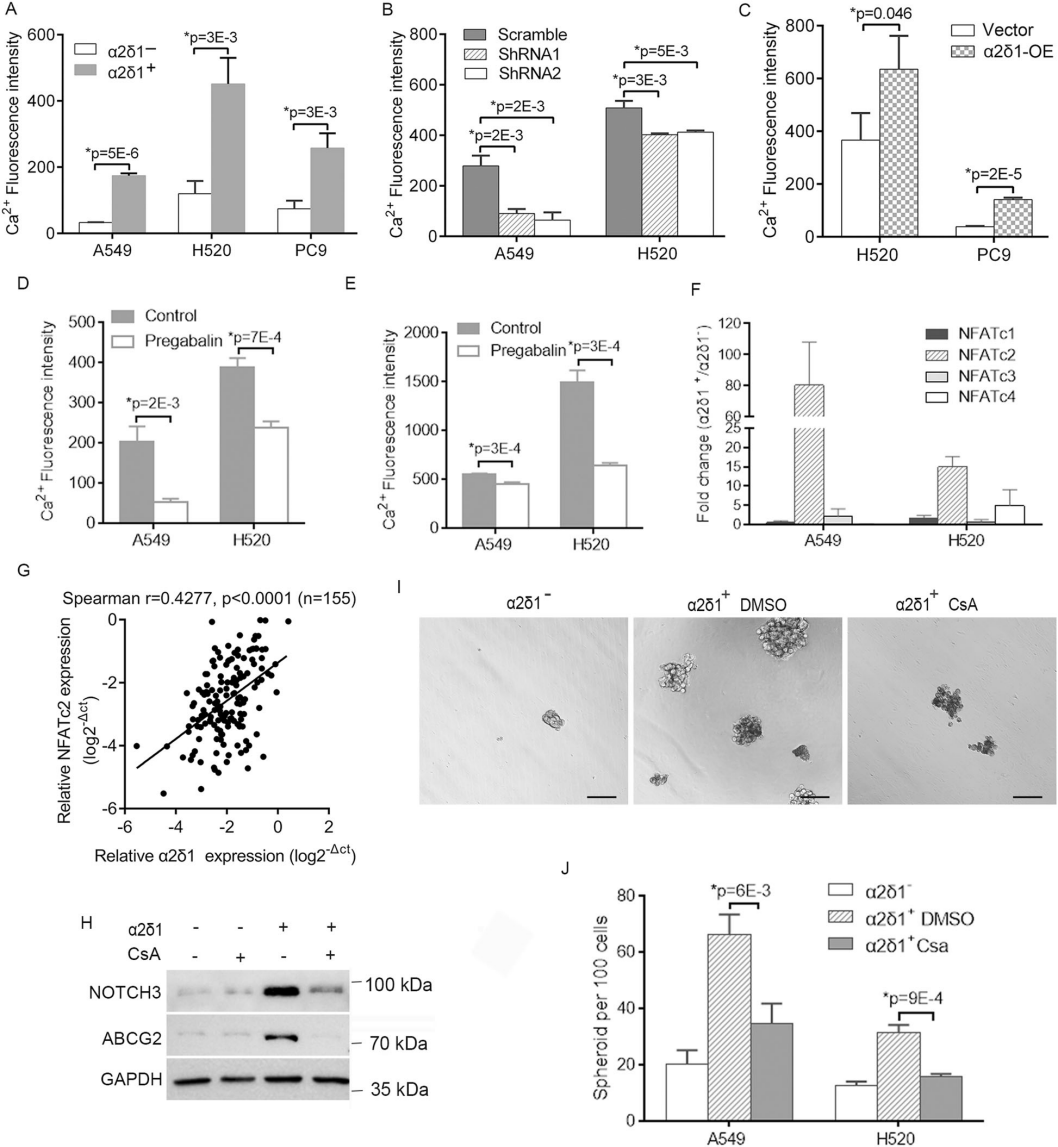
Ca^2+^/NFATc2 is responsible for α2δ1-mediated TIC properties. **A**–**C** The [Ca^2+^]_i_ levels were detected by flow cytometry using Ca^2+^ probe Fluo-4/AM in sorted α2δ1^+^ and α2δ1^−^ fractions from indicated cell lines (**A**), α2δ1^+^ cells from indicated sources with α2δ1 knockdown by shRNAs (**B**), and the indicated cells overexpressing α2δ1 (**C**). **D**, **E** The [Ca^2+^]_i_ levels were measured by flow cytometry in α2δ1^+^ A549 and H520 subpopulations (**D**), as well as in A549 and H520 cells overexpressing α2δ1 (**E**) following treatment with pregabalin at 10 μmol/L for 24 h. **F** The expression of NFATc1-4 was measured in sorted α2δ1^+^ and α2δ1^−^ subsets from indicated sources by qRT-PCR. **G** The relationship between the expression of *α2δ1* with *NFATc2* mRNA in clinical samples of NSCLC as detected by qRT-PCR. (**H**) Western blot results demonstrating the effects of cyclosporinA (CsA) on the expression of NOTCH3 and ABCG2 in sorted α2δ1^+^ and α2δ1^−^ subpopulations of A549 cells. GAPDH is an internal loading control. **I** Representative phase-contrast micrographs showing the effects of CsA on the sphere-forming ability of the α2δ1^+^ A549 cells. Scale bar, 100 µm. **J** Histograms showing the sphere-forming efficiencies of the α2δ1^+^ fractions from indicated cell lines treated with DMSO or CsA (1 µmol/L) (*n* = 3). All error bars represent SD of three independent experiments. *Student’s *t*-test.

Since α2δ1 has been reported previously to cause calcium influx into cells by serving as a subunit of voltage-dependent calcium channel^13,28,29^, we measured the intracellular Ca^2+^ levels ([Ca^2+^]_i_) in NSCLC cell lines by flow cytometry to gain insight into the mechanism(s) by which α2δ1 upregulates NOTCH3 and subsequently promotes the properties of α2δ1^+^ TICs. Significantly higher levels of [Ca^2+^]_i_ were detected in the α2δ1^+^ cells purified from A549, H520, and PC9 cell lines than in their negative counterparts (Fig. 6A). Knockdown of α2δ1 in purified α2δ1^+^ A549 and H520 cells with shRNAs resulted in remarkable decrease of [Ca^2+^]_i_, while forced expression of α2δ1 in H520 and PC9 cells led to significantly opposite effect (Fig. 6B, C). As additional evidence to support that α2δ1 was functionally involved in the influx of Ca^2+^ into the cells of α2δ1^+^ NSCLC TICs, the levels of [Ca^2+^]_i_ could be inhibited significantly in sorted α2δ1^+^ A549 and H520 cells, as well as A549 and H520 cells overexpressing α2δ1 when these cells were treated with pregabalin, a well-characterized inhibitor of α2δ1 (Fig. 6D, E).

We then tested if elevated [Ca^2+^]_i_ could activate the NFAT signaling pathway in α2δ1^+^ NSCLC TICs. Of the 4 NFATc isoforms, the isoform *NFATc2* was found expressed at a much higher level in purified α2δ1^+^ fractions from both the A549 and H520 cell lines than their α2δ1^−^ populations as demonstrated by qRT-PCR (Fig. 6F). Furthermore, the expression of *NFATc2* mRNA was positively correlated with *α2δ1* mRNA in clinical NSCLC specimens (Fig. 6G). Subsequently treatment of α2δ1^+^ A549 cells with cyclosporin A(CsA), a calcineurin inhibitor, could dramatically downregulate the expression of stem cell-related genes such as NOTCH3 and ABCG2 (Fig. 6H). Importantly, cyclosporin A could also significantly suppress the spheroid forming frequencies of the α2δ1^+^ subpopulation of A549 and H520 cells (Fig. 6I, J).

All of these data suggest that the roles of α2δ1 in the upregulation of the stem cell-related genes including NOTCH3 and subsequent driving the self-renewal properties of NSCLC TICs are dependent on Ca^2+^-calcineurin-NFATc2 signaling.

### NFATc2 directly activates the expression of ABCG2 and NOTCH3

**Fig. 7:**
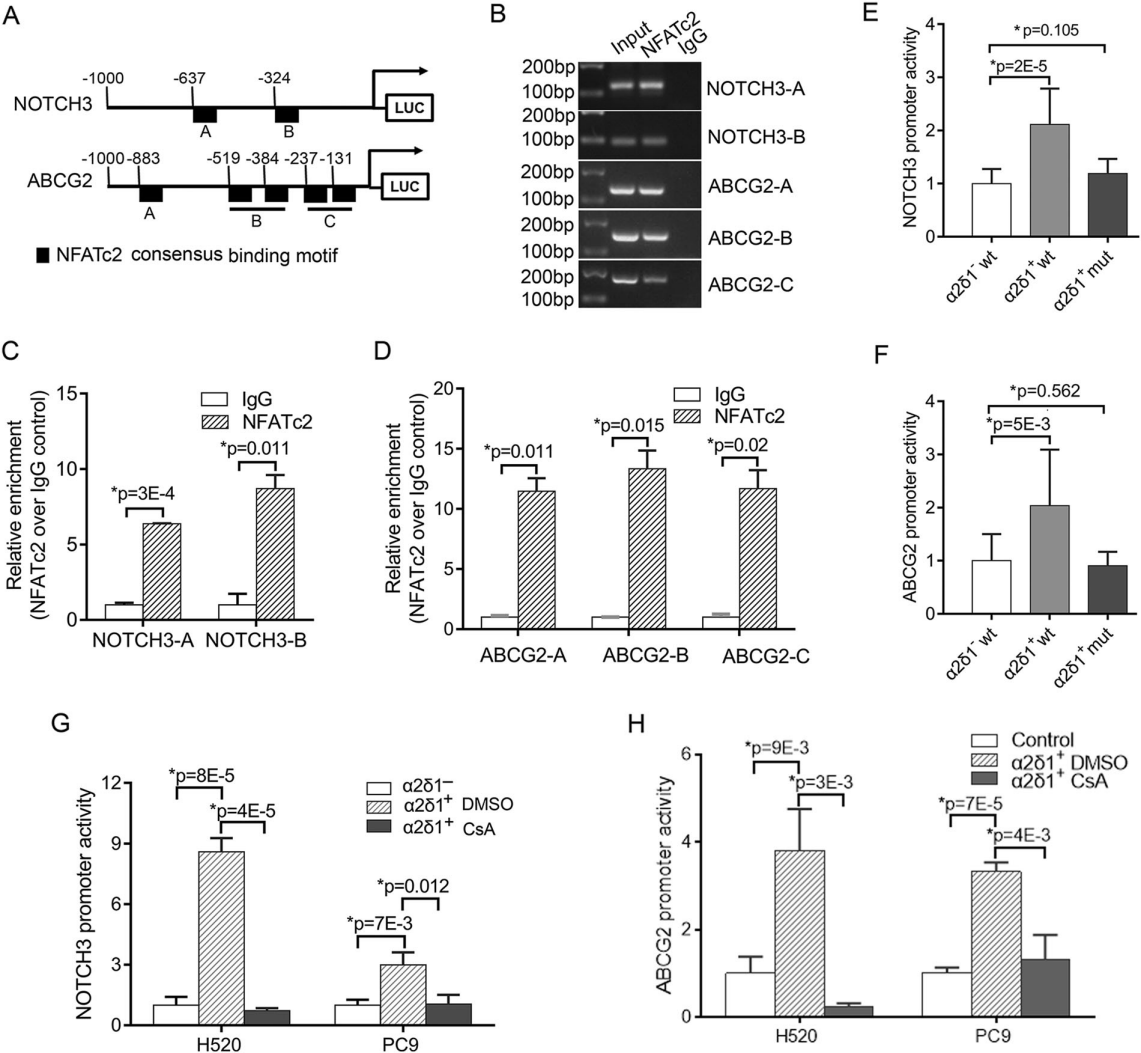
NFATc2 activates the expression of NOTCH3 and ABCG2 by binding to their promoters. **A** Schematic depiction of the NOTCH3 and ABCG2 promoters that are fused to a luciferase reporter gene. **B** ChIP assay was performed using control IgG and anti-NFATc2 antibody in A549 cells. The precipitated DNA was amplified with primer pairs flanking the putative NFATc2-binding regions of indicated promoters as depicted in (**A**) by PCR. **C**, **D** Quantitative analysis of NFATc2-binding to the indicated promoter regions of NOTCH3 and ABCG2 was determined by ChIP assay followed by qPCR. **E**, **F** The luciferase activities of NOTCH3 and ABCG2 reporters in the indicated cell fractions. The indicated reporters with wild type or mutant NFATc2-binding consensus sequences were co-transfected with PRL-TK plasmid, which serves as an internal control, into sorted α2δ1^−^ and α2δ1^+^ A549 cells. Luciferase values were normalized to Renilla reporter activities. **G**, **H** The luciferase activities of wild type NOTCH3 and ABCG2 reporters in the indicated cell fractions treated with control vehicle DMSO or CsA (1 µmol/L). All data are presented as mean ± SD of three independent experiments (*n* = 3). *Student’s *t*-test.

To further test if NFATc2 transcriptionally upregulates the expression of stem cell-related genes of α2δ1^+^ TICs, we performed NFATc2 binding site analysis in the promoters of ABCG2 and NOTCH3 using JASPAR online software. There were a total of 2 and 5 presumptive NFATc2 binding sites found within the region from −1000 to 1 bp bases in the promoters of NOTCH3 and ABCG2, respectively (Fig. 7A). The occupancies of NFATc2 at the promoters of ABCG2 and NOTCH3 were then validated by PCR following ChIP assay (Fig. 7B). Significant binding of NFATc2 to the promoters of ABCG2 and NOTCH3 was revealed with more than a five-fold increase of precipitated target DNA (Fig. 7C, D).

To determine whether these NFATc2 sites were involved in the activation of the promoters of ABCG2 and NOTCH3, we generated the luciferase reporter constructs driven by ABCG2 or NOTCH3 promoters containing either the wild type or the mutant NFATc2 binding sites. Their activities were tested in purified α2δ1^+^ and α2δ1^−^ subsets of A549 cells. The reporter activities of both the wild type NOTCH3 and ABCG2 promoters were dramatically higher in the α2δ1^+^ subpopulation than those in the respective α2δ1^−^ subset, and their activities in α2δ1^+^ cells could be inhibited by CsA treatment, whereas the activities of those reporters containing mutant NFAT2c binding sites showed no significant difference in both the α2δ1^+^ and α2δ1^−^ subpopulation (Fig. 7E–H).

These data collectively confirmed that the direct binding of NFATc2 to the promoters of ABCG2 and NOTCH3 is responsible for the activation of the expression of these two genes in α2δ1^+^ TICs of NSCLC.

### Anti-α2δ1 antibody retards the growth of NSCLC in vivo and eradicates TICs

**Fig. 8:**
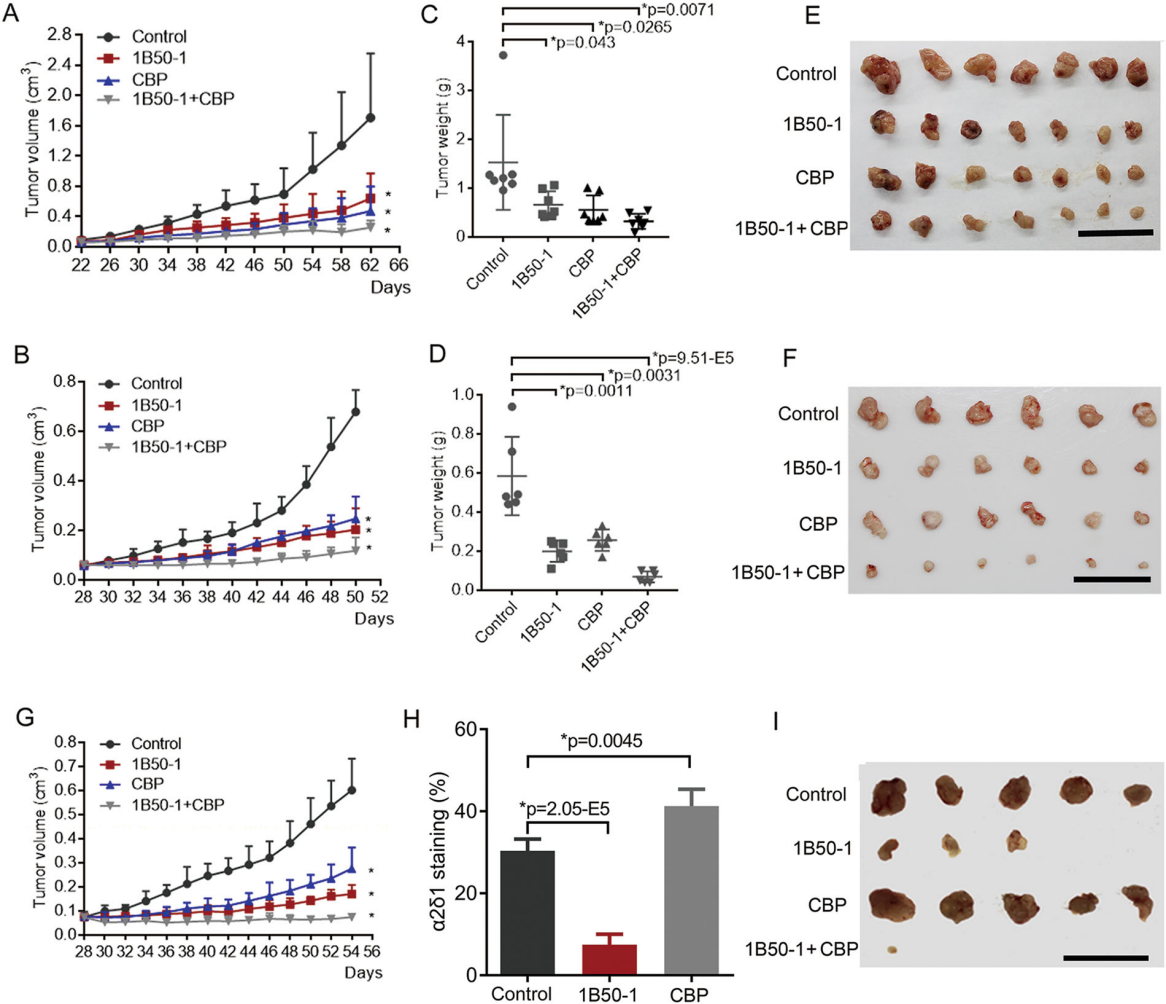
The therapeutic effects of anti-α2δ1 mAb1B50-1 on established NSCLC engraftments in NOD/SCID mice. **A**, **B** Growth curves of A549 (**A**) and H520 (**B**) engraftments treated with 800 μg/mouse 1B50-1, 1.5 mg/kg carboplatin (CBP), or the combination of both, every other day after the tumors were visible. **C**, **D** The histograms showing the average weight of the dissected tumors of A549 (**C**) and H520 (**D**) engraftments treated with indicated regimens. **E**, **F** Photographs of the dissected tumors of A549 (**E**) and H520 (**F**) engraftments received the indicated treatments. (**G**) Growth curves of one NSCLC PDX tumors i.p. injected with 1B50-1, CBP, or the combination of both. **H** Histogram demonstrates the percentage of α2δ1^+^ cells in the residual tumors of A549 xenografts received the indicated treatment as detected by flow cytometry. **I** Dissected tumors showing the tumor-initiating ability of the A549 engraftment residues after each drug treatment, which was assayed by re-transplanting 10^4^ cells per site with Matrigel into NOD/SCID mice. Scale bars, 5 cm. All error bars indicate SD. *p ≤ 0.05, compared with vehicle control group. Student’s *t*-test.

The above results prompted us to investigate whether blocking the function of α2δ1 with mAb1B50-1 could have a therapeutic effect on NSCLC by eliminating TICs. The therapeutic effect of 1B50-1 on established engraftments of the cell lines A549 and H520 in NOD/SCID mice were tested by administering mAb1B50-1 i.p. alone, or combined with cytotoxic drug carboplatin. As shown in Fig. 8A–F, the growth of both the A549 and H520 xenografts was significantly suppressed with the treatment of mAb1B50-1, by ratios of as many as 56.9% and 65.8%, respectively, which were comparable to those with the treatment of carboplatin, whereas the combinational treatment of α2δ1 mAb with carboplatin resulted in inhibition rates of 79.0% and 88.3% on the A549 and H520 xenografts, respectively. Similar effects were also observed on a patient-derived xenograft model with these treatments (Fig. 8G).

To address if the treatments have any effects on the content of TICs, the percentages of TICs in the residues of the treated A549 xenografts were then detected by flow cytometry. Comparing with the control group treated with control vehicle, the proportion of α2δ1^+^ cells decreased remarkably in the residual tumors treated with anti-α2δ1 antibody alone, whereas the α2δ1^+^ cells were dramatically enriched in those residual tumors treated with carboplatin (Fig. 8H). Subsequent transplantation 10^4^ cells from tumors that received 1B50-1 treatment into recipient mice generated very tiny nodules in three out of five mice, while those dissociated cells from control vehicle and carboplatin-treated tumors formed significantly larger tumors in all the transplanted mice. Interestingly, only a negligible nodule was found in one out of five mice transplanted with residual cells from an engrafted tumor received combined therapy (Fig. 8I). These data suggest that TICs were indeed reduced following anti-α2δ1 antibody therapy in vivo, especially after the combinational treatment.

## Discussion

The identification of functional surface markers that could better define TICs with distinct phenotypes is instrumental for developing efficient strategies of targeted therapies in NSCLC patients. In the present study, we demonstrate that a subpopulation of TICs could be specifically isolated from cell lines and fresh tumor tissues of NSCLC using a mAb against α2δ1. These α2δ1^+^ NSCLC cells are more enriched for TICs than those defined by CD133, or CD166, the two well-recognized surface markers of NSCLC TICs^8,10,12^. Interestingly, α2δ1 was found to be functionally sufficient and indispensable for the acquirement and subsequently maintenance of the stem cell-like properties of these TICs via the influx of calcium into cells, which in turn activates the calcium signaling pathway cascade. Hence, α2δ1 marks a unique subpopulation of TICs of NSCLC with highly activated calcium signaling.

Our current study identified that the role of α2δ1 in driving the stem cell-like properties of NSCLC TICs is Notch signaling dependent. Moreover, α2δ1 was demonstrated to be able to activate the expression of stem cell-related genes such as NOTCH3 and ABCG2 through the Ca^2+^-calcineurin-NFAT pathway. Although the crosstalk between Notch and calcium signaling pathways has been appreciated in a couple of cellular processes^30,31^, to the best of our knowledge, this is the first study linking the role of calcium influx mediated by α2δ1 to Notch signaling activation in the biology of NSCLC TICs, uncovering an unappreciated molecular mechanism on how Notch, a key regulator of many reported TIC population^24,32^, is activated in NSCLC TICs. In addition to its ability to activate the Notch signaling, the Ca^2+^-calcineurin-NFAT pathway could up-regulate the expression of ABCG2, the molecule responsible for multi-drug resistance of TICs. NFAT2c has also been shown to enhance the TIC phenotypes of lung cancer by directly activating the expression of SOX2^33^. Hence, it is likely that both the calcineurin/NFAT and Notch signaling pathways work coordinately to determine the stem cell-like properties of NSCLC TICs.

Here, we also identified that α2δ1 is an amenable therapeutic target for the treatment of lung cancer. Both silencing of α2δ1 and treatment with α2δ1 blocking antibody 1B50-1 could dramatically retard the tumorigenicity of NSCLC TICs, and the therapeutic effects of 1B50-1 on NSCLC were attributed to its ability to eradicate TICs in the tumors as shown by loss of serial transplantation capacity in NOD/SCID mice after treatment. Moreover, the therapeutic effects of 1B50-1 could be further augmented by the addition of carboplatin, supporting that a combinational therapy strategy in which TICs and tumor-mass constituent non-TICs are simultaneously targeted is required to achieve an efficient therapy outcome in solid tumors as demonstrated in many other studies^34,35^. Most importantly, α2δ1 controls the expression of Notch, the therapy with α2δ1 blocking antibody 1B50-1 hence targets the upstream of Notch signaling pathway, and therefore targeted therapy against α2δ1 represents a more efficient way of therapy against TICs than those targeted Notch signaling pathway itself. Finally, this study, along with our previous work on hepatocellular carcinoma^13^, demonstrate that targeting the calcium channel α2δ1 subunit with 1B50-1 to prevent calcium influx is a novel cancer therapeutic strategy for targeted therapy against TICs.

In conclusion, the voltage-gated calcium channel α2δ1 subunit is a novel functional marker and therapeutic target for NSCLC TICs. By using this marker, a subset of NSCLC TICs with stem cell-like properties, activated calcium and Notch signaling can be identified at the highest TIC frequency. Furthermore, α2δ1 plays essential roles in the acquirement and subsequent maintenance of the stem cell-like properties of NSCLC TICs through Ca^2+^ -NFAT mediated activation of Notch pathway. Most importantly, the mAb1B50-1, which blocks the function of α2δ1, has remarkably therapeutic effects on NSCLC xenografts, providing a novel therapeutic approach targeting TICs for NSCLC patients.

## Materials and methods

### Cell lines and clinical samples

Human NSCLC cell lines A549, H520, H292, and H1299 were originated from the American Type Culture Collection (ATCC), and PC9, H157 and GLC82 cell lines were obtained from Deutsche Sammlung von Mikroorganismen und Zellkulturen (DSMZ). These cells were cultured in RPMI 1640 medium supplemented with 10% fetal bovine serum (FBS), 100 units/mL penicillin and 100 µg/mL streptomycin (Invitrogen, Grand Island, NY, USA) in a humidified 5% CO_2_ incubator at 37 °C. The cell lines were authenticated using polymorphic short tandem repeat loci analysis and were cleared off mycoplasma contamination.

The primary NSCLC and matched normal tissue samples were collected from patients who had undergone complete resection (R0) at Peking University Cancer Hospital with written informed consent. Some of the fresh primary NSCLC tissues were minced and digested with DNase and collagenase/dispase, followed by transplantation into NOD/SCID mice to generate PDX model.

### Immunofluorescent staining and flow cytometry

The single-cell suspension from NSCLC cell lines, tumor tissues were prepared according to our published protocol^13^, and were immediately incubated with the antibodies including mAb1B50-1 against α2δ1^13^ conjugated with fluorescein isothiocyanate (FITC) using BD Lightning conjugation kits (Expedeon Ltd., Cambridge, UK), CD24- allophycocyanin (APC), CD90-APC, EpCAM-FITC and CD133-allophycoyanin (PE) obtained from Miltenyi Biotech GmbH (Bergisch Gladbach, Germany); and CD166-PE purchased from BD Biosciences (Bedford, MA, USA). To measure Ca^2+^ flux level, the cells were incubated with Fluo-4/AM (Invitrogen). After filtering through a 40-μm nylon mesh, the viable and single cells were gated for analyses or sorting on a FACSAria II flow cytometer (BD Biosciences). The isotype control was used as reference and data were processed using FlowJo VX software.

### Tumorigenicity assay

Female 5- to 6-week-old NOD/SCID mice (Vitalriver, Beijing, China) were used for the studies following protocols approved by the Peking University Cancer Hospital Animal Care and Use Committee. To test the tumorigenic potential, cells were suspended in 50 μl of 1:1 mix of serum-free medium and Matrigel (BD Biosciences), and were injected s.c. into the armpit of mice. Tumor formation was monitored weekly. For the therapeutic assay, 2 × 10^6^ cells per mouse were s.c. transplanted into the frank of mice. When all the tumors were visible, randomly separated groups of mice with comparable sizes of tumors were administered intraperitoneally (i.p.) with control vehicle, anti-α2δ1 1B50-1 mAb (800 μg/mice), carboplatin (40 mg/kg), or the combination of both every other day. The tumors were measured in two dimensions with calipers and individual tumor volumes (*V*) were calculated by the formula: *V* = (length X width^2^)/2. The sample sizes of animals were determined by calculations derived from our experience. No sample was excluded from the analyses, and the investigators were not blinded to the group allocation during the experiment and outcome assessment.

### Immunohistochemistry staining

Sections of frozen NSCLC tissues were fixed with methanol for 30 s. After blocking with 5% non-fat milk in PBS, slides were incubated with mAb1B50-1 at 4 °C overnight, followed by incubation with FITC-goat anti-mouse IgG. Nuclei were stained with 4,6-diamidino-2-phenylindole dihydrochloride (DAPI; Polysciences, Warrington, PA) at 0.5 μg/ml. Specimens were mounted in 90% glycerol/PBS containing 2.5% 1, 4-diazabicyclo(2,2,2)octane. Slides were examined with a Leica SP5 confocal microscope (Leica, Wetzlar, Germany).

### RNA preparation and quantitative real-time PCR (qRT-PCR)

Total RNA was extracted from the NSCLC cell lines and specimens using the RNAeasy Kit (Qiagen, Valencia, CA) according to the manufacturer’s instruction. Complementary DNA was synthesized from 2 μg total RNA using the Multiscribe reverse transcriptase (Invitrogen, Grand Island, NY, USA). Quantitative PCR was performed using SYBR Green PCR Master Mix (Roche, Mannheim, Germany) on LightCycler® 480 Real-Time PCR System (Roche). The glyceraldehyde-3-phosphate dehydrogenase (GAPDH) was used as an internal control. All primer sequences were listed in Supplementary Table 6. The relative gene expression for each sample was calculated using the 2^−ΔΔCt^ method^36^.

### Western blot

Cells were homogenized in radioimmunoprecipitation assay buffer supplemented with Complete Mini protease inhibitor cocktail, and phosphatase inhibitor cocktail (Roche, Mannheim, Germany). Equal amounts of protein were resolved by SDS-PAGE, and Western blot were carried out following standard protocols. The primary antibodies used were given in Supplementary Table 7. The HRP-conjugated secondary antibodies were purchased from Jackson ImmunoResearch Laboratories Inc. (West Grove, PA, USA). Immuno-complexes were visualized with ImmobilonTM Western Chemiluminescent HRP substrate (Millipore, Billerica, MA, USA).

### Apoptosis assay

Apoptotic assay was performed in sorted α2δ1^+^ and α2δ1^−^ cells treated with carboplatin by FITC Annexin V Apoptosis Detection Kit (BD Biosciences) according to the manufacturer’s instruction. In brief, suspension of treated cells was incubated with FITC Annexin V and propidium iodide for 20 min on ice, followed by analysis using BD Accuri™ C6 flow cytometer (BD Biosciences).

### Sphere formation assay

Sphere formation assay was carried out in ultralow attachment 96-well plates (Corning Incorporated Life Sciences, Acton, MA, USA) by plating 100 cells per well in DMEM/F-12 serum-free medium containing B27, 20 ng/ml epidermal growth factor, 20 ng/ml basic fibroblast growth factor (Invitrogen), 1% methylcellulose (Sigma-Aldrich, St. Louis, MO, USA). After 2–3 weeks of cultivation, the formed spheroids were counted under an Axio Observer A1 inverted microscope (Carl Zeiss Microscopy GmbH, Jena, Germany).

### Chromatin immunoprecipitation (ChIP) assay

ChIP assay was done with the ChIP Assay kit (Millipore) following the vendor’s instruction. In brief, cells were cross-linked with 1% formaldehyde at 37 °C for 10 min, followed by incubation with 150 mM glycine for 5 min at room temperature to stop cross-linking. The cells were then harvested in SDS lysis buffer containing protease inhibitors. After the lysates were sonicated to shear the DNA into fragments of 200–1000 bps, immunoprecipitation with NFATc2 (Abcam) or nonspecific IgG was carried out overnight at 4 °C. Protein G Sepharose (GE Healthcare, Little Chalfont, Buckinghamshire, UK) was added to the suspension to collect the antibody/NFATc2/DNA complex, followed by rinsing with each of Low Salt Immune Complex Wash Buffer, High Salt Immune Complex Wash Buffer, LiCl Immune Complex Wash Buffer, and TE Buffer. The precipitated products were then eluted with elution buffer containing 1% SDS, 0.1 mol/L NaHCO_3_, reversed the NFATc2/DNA cross-link with 5 mol/L NaCl at 65°C for 4 h. The DNA was finally eluted, and purified by PCR Purification Kit from Qiagen. PCR was performed with primers listed in Supplementary Table 8.

### Vector construction

The construction of α2δ1 overexpression and shRNA lentiviral vectors, packaging of lentiviruses and infection of cells were the same as described in our previous paper^13^. For luciferase reporter construction, the presumptive promoters of human NOTCH3 and ABCG2 containing the wild type NFATc2 binding consensus sequences (5′-TTTCC-3′) or the mutant sequences (5′-CAGCC-3′) were synthesized by Sangon Biotech Co. (Shanghai, China) and subcloned into pGL3-basic vector.

### Luciferase reporter assay

Luciferase reporter assay was done according to the manufacturer’s protocol of Dual-Luciferase Reporter Assay system (Promega, Madison, USA). In brief, the cells were plated in 96-well culture plates and transfected with the indicated constructs using lipofectamine 3000 (Invitrogen). Luciferase activity was measured using the Lumat LB 9501 Luminometer (Berthold Technologies). Firefly luciferase values were normalized to Renilla luciferase value, yielding relative activities and are shown as mean values ± SD.

### Statistics

Data were analyzed using SPSS software (version 16.0, SPSS Inc., Chicago, IL), and the significance of differences was determined with a double-sided Student’s *t*-test or one-way ANOVA unless otherwise specified. TIC frequencies were determined using the ELDA webtool at http://bioinf.wehi.edu.au/software/elda/ based on extreme limiting dilution analysis^37^. A *p* ≤ 0.05 was considered statistically significant.

## Supplementary information

Supplementary Table 1

Supplementary Table 2

Supplementary Table 3

Supplementary Table 4

Supplementary Table 5

Supplementary Table 6

Supplementary Table 7

Supplementary Table 8
