# Supplementary material for: Calcium channel α2δ1 subunit is a functional marker and therapeutic target for tumor-initiating cells in non-small cell lung cancer
Source: Cell Death Dis. 2021 Mar 11;12(3):257. doi: 10.1038/s41419-021-03522-0 (PMC7952379; doi:10.1038/s41419-021-03522-0)
Supplement: Supplementary file 1 — Supplementary Table 1 [file 41419_2021_3522_MOESM1_ESM.docx]

Supplementary Table 1: The percentage of positive cells of indicated molecules in NSCLC cell lines

| Cells | CD24 | CD90 | EpCAM | α2δ1 | CD133 | CD166 |
| --- | --- | --- | --- | --- | --- | --- |
| A549 | 43.53±1.07 | 1.5±0.41 | 1.88± 0.21 | 29.08±4.83 | 3.57±1.87 | 70.47±2.74 |
| H520 | 34.57±3.09 | 1.26±0.11 | 92.33±5.03 | 3.41±0.57 | 4.76±1.1 | 29.11±5.3 |
| H292 | 92.27±2.31 | 1.3±0.31 | 97.17±1.08 | 5.94±0.36 | 2.24±0.74 | 21.8±4.77 |
| H1299 | 1.97±0.56 | 1.0±0.19 | 1.43±0.09 | 9.2±1.26 | 1.82±0.64 | 6.14±1.64 |
| PC9 | 3.51±1.16 | 1.63±0.31 | 74.13±3.0 | 1.58±0.17 | 5.34±1.64 | 29.11±5.32 |
| H157 | 0.94±0.06 | 0.82±0.1 | 1.45±0.16 | 2.32±0.53 | 1.39±0.1 | 6.71±0.63 |
| GLC82 | 23.07±3.2 | 1.06±0.06 | 14.63±1.16 | 1.82±0.15 | 1.98±0.59 | 9.77±3.1 |

Data are means ± S.D.
