# Supplementary material for: Calcium channel α2δ1 subunit is a functional marker and therapeutic target for tumor-initiating cells in non-small cell lung cancer
Source: Cell Death Dis. 2021 Mar 11;12(3):257. doi: 10.1038/s41419-021-03522-0 (PMC7952379; doi:10.1038/s41419-021-03522-0)
Supplement: Supplementary file 2 — Supplementary Table 2 [file 41419_2021_3522_MOESM2_ESM.docx]

Supplementary Table 2: The tumorigenicity of each fraction of A549 cells in NOD/SCID mice

| Markers | Tumor formation | | | Frequency of tumorigenic cells (95% CI) | P value |
| --- | --- | --- | --- | --- | --- |
|  | 1000 | 100 | 50 |  |  |
| CD24^−^ | 2/5 | 0/5 | 0/5 | 1/2340(1/9266-1/591) | 0.499 |
| CD24^+^ | 1/5 | 0/5 | 0/5 | 1/5234 (1/36830-1/744) |  |
| CD90^−^ | 2/5 | 1/5 | 0/5 | 1/1530 (1/4946-1/474) | 0.12 |
| CD90^+^ | 4/5 | 2/5 | 0/5 | 1/504 (1/1269-1/200) |  |
| EpCAM^−^ | 0/5 | 0/5 | 0/5 | Infinity (Infinity -1/1919) | 0.0276 |
| EpCAM^+^ | 2/5 | 1/5 | 0/5 | 1/1530 (1/4946-1/474) |  |
| α2δ1^−^ | 1/5 | 1/5 | 0/5 | 1/2584 (1/10830-1/616) | 1.13e-07 |
| α2δ1^+^ | 5/5 | 4/5 | 3/5 | 1/58.6 (1/131-1/26) |  |
