# Supplementary material for: Calcium channel α2δ1 subunit is a functional marker and therapeutic target for tumor-initiating cells in non-small cell lung cancer
Source: Cell Death Dis. 2021 Mar 11;12(3):257. doi: 10.1038/s41419-021-03522-0 (PMC7952379; doi:10.1038/s41419-021-03522-0)
Supplement: Supplementary file 3 — Supplementary Table 3 [file 41419_2021_3522_MOESM3_ESM.docx]

Supplementary Table 3: Association of α2δ1 mRNA expression with clinicopathologic characteristics in 169 cases of NSCLC patients

| Variable | Case  number (n) | α2δ1 expression (2^-△Ct^) | | |
| --- | --- | --- | --- | --- |
|  |  | Median | Range | P value ^a^ |
| Gender |  |  |  | 0.575 |
| Male | 122 | 0.0118 | 0.000188-1.46 |  |
| Female | 47 | 0.0152 | 0.000315-2.884 |  |
| Age (year) |  |  |  | 0.706 |
| < 60 | 74 | 0.0135 | 0.000268-2.88 |  |
| ≥ 60 | 95 | 0.0143 | 0.000188-2.21 |  |
| Smoking |  |  |  | 0.483 |
| No | 58 | 0.0134 | 0.000315-2.88 |  |
| Yes | 111 | 0.0177 | 0.000003-2.21 |  |
| Differentiation |  |  |  | 0.666 |
| Well | 28 | 0.0183 | 0.00027-2.56 |  |
| Moderate | 69 | 0.0151 | 0.00019-2.28 |  |
| Poor | 56 | 0.0104 | 0.00029-2.88 |  |
| Undifferentiated | 16 | 0.0103 | 0.00032-0.322 |  |
| Histological type |  |  |  | 0.287 |
| Adenocarcinoma | 62 | 0.0094 | 0.00029-2.56 |  |
| Squamous Carcinoma | 92 | 0.0179 | 0.00019-2.88 |  |
| Adenosquamous | 10 | 0.083 | 0.0027-0.89 |  |
| Large Cell Carcinoma | 5 | 0.0046 | 0.00027-0.019 |  |
| Venous invasion |  |  |  | 0.199 |
| Absent | 134 | 0.0118 | 0.000268-2.884 |  |
| Present | 35 | 0.0313 | 0.000188-2.283 |  |
| Metastasis |  |  |  | 0.027 |
| Absent | 111 | 0.011 | 0.000268-2.556 |  |
| Present | 58 | 0.019 | 0.000188-2.884 |  |
| TNM stage^b^ |  |  |  | 0.015 |
| Early stage I-II | 99 | 0.0122 | 0.000322-2.884 |  |
| Advanced stage III-IV | 70 | 0.0156 | 0.000188-2.283 |  |
| Survival (year) |  |  |  | 0.015 |
| < 4 | 106 | 0.25 | 0.000188-2.884 |  |
| ≥ 4 | 63 | 0.0074 | 0.00029-2.1 |  |
| Recurrence |  |  |  | 0.001 |
| Absent ^c^ | 98 | 0.0096 | 0.000268-2.556 |  |
| Present ^d^ | 71 | 0.271 | 0.000188-2.884 |  |

^a^ Mann-Whitney test.

^b^ According to the International Union Against Cancer and the American Joint Committee on Cancer (UICC/AJCC) TNM staging system

^c^ Recurrence within 2 years of diagnosis.

^d^ No recurrence within 4 years of diagnosis.
