# Supplementary material for: Calcium channel α2δ1 subunit is a functional marker and therapeutic target for tumor-initiating cells in non-small cell lung cancer
Source: Cell Death Dis. 2021 Mar 11;12(3):257. doi: 10.1038/s41419-021-03522-0 (PMC7952379; doi:10.1038/s41419-021-03522-0)
Supplement: Supplementary file 4 — Supplementary Table 4 [file 41419_2021_3522_MOESM4_ESM.docx]

Supplementary Table 4: Cox regression analysis of α2δ1 mRNA expression and clinicopathologic characteristics in 169 cases of NSCLC patients

| Variable | Case (n) | Multivariate analysis ^a^ | |
| --- | --- | --- | --- |
|  |  | Relative Risk (95% CI ^d^) | P value |
| Gender |  |  | 0.617 |
| Male | 122 |  |  |
| Female | 47 | 0.873 (0.513-1.487) |  |
| Age (year) |  |  | 0.715 |
| < 60 | 74 |  |  |
| ≥ 60 | 95 | 0.917 (0.574-1.463) |  |
| Venous invasion |  |  | 0.098 |
| Absent | 134 |  |  |
| Present | 35 | 1.549 (0.923-2.602) |  |
| Metastasis |  |  | 0.047 |
| Absent | 111 |  |  |
| Present | 58 | 1.607 (0.997-2.59) |  |
| TNM stage ^b^ |  |  | 0.006 |
| Early stage I-II | 99 |  |  |
| Advanced stage III-IV | 70 | 1.935 (1.21-3.097) |  |
| α2δ1 ^c^ |  |  | 0.003 |
| Low | 84 |  |  |
| High | 85 | 2.077 (1.274-3.388) |  |

^a^ Cox regression analysis; ^b^ UICC/AJCC TNM staging system; ^c^ the cases are divided into low and high group according to the median level of α2δ1 mRNA expression;

^d^ CI, Confidence interval.
