# Supplementary material for: Calcium channel α2δ1 subunit is a functional marker and therapeutic target for tumor-initiating cells in non-small cell lung cancer
Source: Cell Death Dis. 2021 Mar 11;12(3):257. doi: 10.1038/s41419-021-03522-0 (PMC7952379; doi:10.1038/s41419-021-03522-0)
Supplement: Supplementary file 5 — Supplementary Table 5 [file 41419_2021_3522_MOESM5_ESM.docx]

Supplementary Table 5: The tumorigenicity of α2δ1^+^, CD133^+^, and CD166^+^ A549 cells

| Makers | Tumor formation | | | Frequency of tumorigenic  Cells (95% CI) | P value |
| --- | --- | --- | --- | --- | --- |
|  | 1000 | 100 | 50 |  |  |
| α2δ1^+^ | 5/5 | 3/5 | 3/5 | 1/81(1/189-1/35) |  |
| CD133^+^ | 2/5 | 2/5 | 1/5 | 1/888 (1/2411-1/327) | 1.76E-04 |
| CD166^+^ | 3/5 | 2/5 | 2/5 | 1/520 (1/1312-1/207) | 2.41E-0 |
