# Supplementary material for: Calcium channel α2δ1 subunit is a functional marker and therapeutic target for tumor-initiating cells in non-small cell lung cancer
Source: Cell Death Dis. 2021 Mar 11;12(3):257. doi: 10.1038/s41419-021-03522-0 (PMC7952379; doi:10.1038/s41419-021-03522-0)
Supplement: Supplementary file 6 — Supplementary Table 6 [file 41419_2021_3522_MOESM6_ESM.docx]

| Supplementary Table 6. Primer sequences for qRT-PCR | |
| --- | --- |
| **Name Sequence** | |
| CACNA2D1 | PF: 5'ACAGCAAGTGGAGTCAATCA |
|  | PR: 5'ACTGCTGCGTGCTGATAAGA |
| NOTCH1 | PF: 5'CCTGAGGGCTTCAAAGTGTC |
|  | PR: 5' CGGAACTTCTTGGTCTCCAG |
| NOTCH3 | PF: 5'GTGTGTGTCAATGGCTGGAC |
|  | PR: 5'GTGACACAGGAGGCCAGTCT |
| NFATc1 | PF: 5'TCTGCTGTTCTCATGGATGC |
|  | PR: 5' TCAGGACTGGTCTTCCATATC |
| NFATc2 | PF: 5'CATCAACGCCCTGACCAC |
|  | PR: 5'GTGGTGCCCAGGTCTTCC |
| NFATc3 | PF: 5'TAATATGTCAGCGAGTATTG |
|  | PR: 5'GATCCAGTCACAATCATTTC |
| NFATc4 | PF: 5'GAGCAGCTGGAGCTGAGG |
|  | PR: 5'TGTAGCCTAGGAGCTTGAC |
| GAPDH | PF: 5'GACCCCTTCATTGACCTCAAC |
|  | PR: 5'CTTCTCCATGGTGGTGAAGA |
