# Supplementary material for: Calcium channel α2δ1 subunit is a functional marker and therapeutic target for tumor-initiating cells in non-small cell lung cancer
Source: Cell Death Dis. 2021 Mar 11;12(3):257. doi: 10.1038/s41419-021-03522-0 (PMC7952379; doi:10.1038/s41419-021-03522-0)
Supplement: Supplementary file 7 — Supplementary Table 7 [file 41419_2021_3522_MOESM7_ESM.docx]

| Supplementary Table 7: Antibodies used in Western blots | | | |  |
| --- | --- | --- | --- | --- |
| Name. | Vender | Cat No. | Species | Dilution |
| CACNA2D1 | Abcam | ab2864 | Mouse monoclonal IgG | 1:2000 |
| NANOG | CST | 4903 | Rabbit monoclonal IgG | 1:1000 |
| SOX2 | CST | 3579 | Rabbit monoclonal IgG | 1:2000 |
| OCT4 | Abcam | ab109183 | Rabbit monoclonal IgG | 1:2000 |
| ABCG2 | Abcam | ab108312 | Rabbit monoclonal IgG | 1:2000 |
| NOTCH1 | CST | 3608 | Rabbit monoclonal IgG | 1:1000 |
| NOTCH3 | CST | 3446, | Rabbit monoclonal IgG | 1:1000 |
| GAPDH | Bioworld | AP0063 | Rabbit monoclonal IgG | 1:100000 |
