# Supplementary material for: Calcium channel α2δ1 subunit is a functional marker and therapeutic target for tumor-initiating cells in non-small cell lung cancer
Source: Cell Death Dis. 2021 Mar 11;12(3):257. doi: 10.1038/s41419-021-03522-0 (PMC7952379; doi:10.1038/s41419-021-03522-0)
Supplement: Supplementary file 8 — Supplementary Table 8 [file 41419_2021_3522_MOESM8_ESM.docx]

Supplementary Table 8. Primer sequences for ChIP-PCR

| **Name Sequence** | |
| --- | --- |
| NOTCH3- Site 1 | PF: 5'AAGTGCAACCTCAGCCTCCT |
|  | PR: 5'GAAACCCGGACTCTACTGAAAA |
| NOTCH3- Site 2 | PF: 5'ATTTGTGCGTCCGTGGCTGT |
|  | PR: 5'CTGGAGCCGGGACAAAAAAC |
| ABCG2- Site A | PF: 5'ATCACTATTTTATTGTTGGGGAAGG |
|  | PR: 5'TGTCACCCAGGCTGGAGTTAAATAG |
| ABCG2*-* Site B | PF: 5'GAGAGAGAGAGAGAGAAATGCCTAT |
|  | PR: 5'AAACAACCAAGCAGGACTATGACAT |
| ABCG2*-* Site C | PF: 5'GTTTACGCACAGAGCAAAGCCA |
|  | PR: 5'CCTCTTCTGGCATTCCTAGCAA |
